# Field notes from the Nigeria Centre for Disease Control 2019 pilot internship program for resident doctors

**DOI:** 10.11604/pamj.2020.35.88.20583

**Published:** 2020-03-25

**Authors:** Oluwatomi Funbi Iken, Kelly Elimian, Chinwe Ochu, Chikwe Ihekweazu

**Affiliations:** 1 University College Hospital, Ibadan, Nigeria; 2 Nigeria Center for Disease Control, Abuja, Nigeria

**Keywords:** Epidemiology, Nigeria Center for Disease Control, resident doctors

## Abstract

The 10-week internship for the pilot cohort of resident doctors from various teaching hospitals in Nigeria was a very rewarding experience. The internship was a beautiful immersion into field epidemiology, rumor surveillance, risk communication, digital tools for surveillance, developing strategic documents, line lists interpretation, weekly presentations and outbreak response coordination alongside working briefly as an incident manager for the Yellow Fever technical working group. Some of the learning points included: meeting coordination, contributions to ongoing research, review of training documents for surveillance officers and the mechanisms of escalating and de-escalating technical working groups in the face of outbreaks and working as an incident manager. There is the need to continue this internship to strengthen the capacity of our emerging health workforce in residency training to address our public health priorities in Nigeria.

## Commentary

The Nigeria Centre for Disease Control (NCDC) was established in 2011 in response to the challenges of public health emergencies and to enhance Nigeria's preparedness and response to epidemics through prevention, detection and control of communicable and non-communicable diseases. Its core mandate is to detect, investigate, prevent and control diseases of national and international public health importance. The mission for the NCDC next five years (2017-2021) is 'to protect the health of Nigerians through evidence-based prevention, integrated disease surveillance and response activities, using a one health approach, guided by research and led by a skilled workforce'. The core functions of the NCDC include: prevent, detect, and control diseases of public health importance, coordinate surveillance systems to collect, analyse and interpret data on diseases of public health importance, support States in responding to small outbreaks, and lead the response to large disease outbreaks, develop and maintain a network of reference and specialized laboratories, lead Nigeria's engagement with the international community on diseases of public health relevance and to conduct, collate, synthesize and disseminate public health research to inform policy. The Centre has close to two hundred staff working across its locations at the Headquarters and the National Reference Laboratory in Abuja, as well as the Central Public Health Laboratory in Lagos State which is a campus of the National Reference Laboratory. The Centre is led by a Director General, the members of staff work in six Directorates. These include: Public Health Laboratory Services, Prevention and Programs Coordination, Emergency Preparedness and Response, Surveillance and Epidemiology, Finance and Accounts, Administration and Human Resources.

The internship for resident doctors in community medicine/public health at the NCDC, is the first cohort intended to provide skills and competencies in various aspects of field epidemiology, providing practical experience, latest knowledge, opportunities for collaboration and hands on experience. A training manual and logbook to guide the posting were available as residents rotated through the various departments. The 10-week internship for the pilot cohort of resident doctors from various teaching hospitals in Nigeria was a very rewarding experience. The internship commenced with an introductory week in which all the residents were introduced to the various units and their roles in the organization. Following this, we participated in a week long curriculum review for training disease surveillance officers in Nigeria. This was very engaging and was also a crash course in revising many concepts in epidemiology. The orientation, lectures, unit rotations, participation in technical working groups/emergency operation centers and response pillars strengthened capacity, for competencies learnt in residency training. The internship was a beautiful immersion into field epidemiology, rumor surveillance, risk communication, digital tools for surveillance, developing strategic documents, line lists interpretation, weekly presentations and outbreak response coordination alongside working briefly as an incident manager for the Yellow Fever technical working group. Some of the learning points included: meeting coordination, contributions to ongoing research, review of training documents for surveillance officers and the mechanisms of escalating and de-escalating technical working groups in the face of outbreaks. It was an enlightening, greatly rewarding, educative and practical experience, integrating the background theoretical knowledge with on field exposure. I had the opportunity to prepare and present slides in different meetings, receive refresher trainings for procedures like lumbar puncture, write meeting minutes and reports, was deployed to a University Teaching Hospital in the country to assess their suitability for selection as a Congenital Rubella Assessment sentinel site using the National Checklist. Other activities I actively participated in included calling state epidemiologists for priority diseases using the provided templates, data analysis from national line-lists for some priority diseases, budget preparation and interpretation for a grant and field calls at the Connect Centre. I also was involved in proposal writing, drafted a short communication article and wrote a few topics for the NCDC alphabetical health topics pages. Alongside my colleagues in the cohort, we attended and participated in the Lassa Fever Outbreak Response Technical working team. I joined the Risk Communication Pillar for the outbreak response. I completed several online courses to develop competencies on International Health Regulation, Integrated Disease Surveillance, antimicrobial resistance which are core thematic areas for the NCDC.

Furthermore, I improved my skills by participating in a week-long review of the training curriculum for disease surveillance officers nationwide. I was able to participate in planning for a national training and its logistics and improved on my presentation skills. Thereafter, I participated in the Lassa Fever Response Mid-Review Meeting, learning about case management, the role of partners and risk communication. I was able to learn first-hand, the huge work in the management of cases, deployments of rapid response teams and the logistics, risk communication, its methods and evaluation and the harmonization of data from the laboratories and cases managed for effective planning. The meeting helped review what the international standards are, what was done on the field and the areas that required subsequent encouragement or improvement. This also brought to the fore for me, how each response pillar seemingly works on its own, but how also they seamlessly fit into one goal, to reduce the burden of diseases in communities. The importance of goal setting, leadership and coordination, clear reporting lines, engagement with state public health units and proper community engagement and buy-in with the ability to clearly present work done was my major learning from this activity. Subsequently, I followed the processes involved in the escalation of a technical working group to an emergency operation center and all the workings of an incident manager for Yellow Fever technical working group in which capacity I served for a week. The role of an incident manager in coordination, receiving and synthesizing reports, liasing with partners, collaborating with the various technical pillars and being able to respond promptly to issues of national concern and data reports was a huge learning curve for me. I learnt how to hold meetings before the main meeting, seek clarification, get the buy-in of stakeholders, see how national level data is generated and interpreted for public health action. This role positively impacted me by helping me integrate what I had learnt in my residency training program, with the dynamic nature of field epidemiology and enhanced my managerial and technical capabilities under the supervision of the main incident manager. One of the numerous highlights was my deployment to a teaching hospital in Northern Nigeria to conduct an assessment of their capacity to host a sentinel surveillance site for Congenital Rubella Syndrome. The trip was my first to Northern Nigeria, alongside an NCDC staff. A checklist was used in assessing the hospital with interviews conducted for the relevant heads of the concerned clinical specialties. The responses were collated from all the centers where the assessments were done including mine and the sites are currently working. This particular experience helped me further appreciate at the national level; the conceptualization of research ideas, the development and deployment of tools and human resources, collaborations with partners, deployment of teams to fields, data analysis and subsequent public health action as a continuum. At the end, I made new friends in the field of public health, went to towns I had never been to, ate unfamiliar meals, learnt how to use new digital tools for public health and increased my network. One major lesson for me was that disease surveillance and response is hard work, dynamic and requires collaborations, especially in resource-limited countries like Nigeria. More improvements are required in refining the training manual/logbook to suit the expectations from resident doctors, which should also reflect personal learning objectives. This will enhance a mutually beneficial experience.

## Conclusion

As the NCDC continues alongside other agencies for health security, there is the continued need to build capacity for prevention and response across the different cadres of health workers and this internship amongst other programs at the Centre offers the opportunity to achieve this on a continuous basis.

## Competing interests

The authors declare no competing interests.

